# Spatiotemporal gait parameters and recurrent falls in community-dwelling elderly women: a prospective study

**DOI:** 10.1590/bjpt-rbf.2014.0067

**Published:** 2015

**Authors:** Bruno S. Moreira, Rosana F. Sampaio, Renata N. Kirkwood

**Affiliations:** Departamento de Fisioterapia, Universidade Federal de Minas Gerais (UFMG), Belo Horizonte, MG, Brazil

**Keywords:** falls, elderly, gait, principal component analysis, biplot, physical therapy

## Abstract

**BACKGROUND:**

Falling is a common but devastating and costly problem of aging. There is no consensus in the literature on whether the spatial and temporal gait parameters could identify elderly people at risk of recurrent falls.

**OBJECTIVE:**

To determine whether spatiotemporal gait parameters could predict recurrent falls in elderly women.

**METHOD:**

One hundred and forty-eight elderly women (65-85 years) participated in this study. Seven spatiotemporal gait parameters were collected with the GAITRite(r) system. Falls were recorded prospectively during 12 months through biweekly phone contacts. Elderly women who reported two or more falls throughout the follow-up period were considered as recurrent fallers. Principal component analysis (PCA) and discriminant analysis followed by biplot graph interpretation were applied to the gait parameters.

**RESULTS:**

After 12 months, 23 elderly women fell twice or more and comprised the recurrent fallers group and 110 with one or no falls comprised the non-recurrent fallers group. PCA resulted in three components that explained 88.3% of data variance. Discriminant analysis showed that none of the components could significantly discriminate the groups. However, visual inspection of the biplot showed a trend towards group separation in relation to gait velocity and stance time. PC1 represented gait rhythm and showed that recurrent fallers tend to walk with lower velocity and cadence and increased stance time in relation to non-recurrent fallers.

**CONCLUSIONS:**

The analyzed spatiotemporal gait parameters failed to predict recurrent falls in this sample. The PCA-biplot technique highlighted important trends or red flags that should be considered when evaluating recurrent falls in elderly females.

## Introduction

Falls among older adults are a major public health concern due to their high incidence, substantial morbimortality rate, and high associated healthcare costs^1^. It is estimated that 30 to 60% of community-dwelling elderly people fall each year, with approximately half of them experiencing multiple falls^2^, and that 10 to 20% of these falls result in injury, hospitalization, and/or death^3^. Falls also generate serious psychological and social consequences. Individuals may experience fear of falling, activity restriction, and loss of confidence, which may consequently facilitate further functional decline, depression, and social isolation^4^. Considering these adverse outcomes, it is imperative to proactively identify individuals at risk for falling.

During the last decade, gait velocity has been repeatedly reported as an appealing, quick, inexpensive, and highly reliable tool in research and clinical practice to assess elderly people at a high risk of negative outcomes^5^, such as falls and recurrent falls. However, its capacity to predict recurrent falls has not been consistently observed. Some previous prospective studies have shown that slow gait velocity is associated with recurrent falls in the elderly population^6^^-^ 8. For example, in a community-based prospective study with a 2-year follow-up conducted in 1,016 participants aged 70 years and older, Luukinen et al.^9^ found that slow walking velocity (<0.77m/s) was an independent risk factor for recurrent falls (age- and sex-adjusted odds ratio=1.79, 95%CI=1.06-3.00). Conversely, other prospective studies failed to observe a significant association between gait velocity and future recurrent falls in older adults^10^^-^ 12.

Gait is a complex motor activity with many measurable facets besides velocity that could help to identify individuals with recurrent falls. In a study involving 96 community-dwelling elderly women (72.8±6.2 years), Lord et al.^11^ demonstrated that elderly women who fell on two or more occasions in a one-year prospective period had significantly reduced cadence and increased stance time than those who did not fall or fell on one occasion only. On the other hand, Hill et at.^10^ identified that double support phase was associated with prediction of recurrent falls in a group of 96 healthy, active community-dwelling women aged 70 years and older. Therefore, it remains unclear whether and to what extent gait velocity and other gait parameters predict recurrent falls in the elderly population.

The present study investigated whether gait velocity and other spatiotemporal gait parameters could predict recurrent falls in a group of community-dwelling women aged 65 years or more. The understanding of the relationship between the gait pattern changes and falls can help health professionals and researchers to develop appropriate preventive approaches and therapeutic interventions to reduce the occurrence of falls in the elderly.

## Method

### Participants

One hundred forty-eight elderly women (65-85 years) were recruited on a volunteer basis from the general community of Belo Horizonte, MG, Brazil. The inclusion criteria were women aged 65 years or older, living independently in the community and able to walk without walking-aid devices. The exclusion criteria were cognitive impairment detectable by the Mini-Mental State Examination (MMSE) using the Brazilian cutoff points based on the degree of education^13^, vestibular symptoms, motor sequel due to rheumatic, orthopedic and/or neurological diseases, strong pain in the spine or lower limbs, accentuated postural deviation, severe foot deformity, visual impairment not corrected by lenses, auditory impairment not corrected by hearing aids, and history of fracture and/or surgery in lower limbs in the past two years. The Ethics Committee from Universidade Federal de Minas Gerais (UFMG), Belo Horizonte, MG, Brazil approved this study (protocol 442/08), and written consent was obtained from all participants.

### Measures and procedures

At baseline, sociodemographic and clinical data were collected and psychological and balance measures known to influence falls risk, as described elsewhere, were completed^14^^-^ 16. Anthropometric data and information about physical exercise were also collected. Participants who reported regular aerobic or strength training two or more times a week were considered as active.

Psychological measures included fear of falling evaluated through the question "Are you afraid of falling?", with a yes or no answer and self-efficacy or confidence of avoiding a fall during activities of daily living assessed using the Brazilian version of the Falls Efficacy Scale-International (FES-I)^17^. The FES-I scores range from 16 to 64, with higher scores representing lower self-efficacy for falls or greater concern about falling.

Dynamic balance was assessed with the Timed Up and Go (TUG)^18^. This test quantifies the time taken for an individual to rise from a chair, walk 3 m, turn around, walk back to the chair and sit again^19^. The participants walked as fast and safely as possible. A trial run was first performed for the subjects to become familiarized with the test and then the TUG was recorded twice. The final score was calculated as the mean of the two trials.

### Spatiotemporal gait parameters

Gait parameters (velocity, cadence, step length, base of support, swing time, stance time, and double support time) were collected with a 5.74 m computerized carpet (GAITRite, CIR Systems, USA). Participants were asked to walk on the mat at their usual pace for six trials. The beginning and end of the course were delimited by cones placed 2 m from the edge of the mat for acceleration and deceleration. The following instruction was given: "Please walk to the cone at your usual speed, go!". Data were processed using the GAITRite(r) software version 3.9. Data from all trials were combined and considered as a single test.

### Falls

A fall was defined as unintentionally coming to the ground or some lower level^20^. The loss of consciousness or sudden onset of paralysis resulting in a fall (stroke or epileptic seizure) and falling as a consequence of sustaining a violent blow and high-trauma falls were not considered as falls^20^. Falls were assessed both retrospectively and prospectively. Retrospective falls were assessed during the initial interview. Participants were asked how many times they had fallen in the previous 12 months. During the following year, participants were contacted by phone every 15 days and asked about the occurrence of falls, and in case of falls, the circumstances and consequences were obtained. Telephone calls were conducted by the research assistants who were unaware of the previous fall conditions of the participants. To avoid recall bias, participants with missed phone calls for a 3-month period were excluded from the study. Participants who reported two or more falls during the follow-up were categorized as "recurrent fallers" whereas participants who experienced no falls or only one fall were considered "non-recurrent fallers".

### Statistical analysis

Independent *t* test and Mann-Whitney test with Bonferroni's correction and chi-square test were used to compare baseline characteristics between the recurrent and non-recurrent fallers groups. All the data were analyzed with a significance level of 0.05.

Gait parameters were first standardized and mean-centered to maximize the variance. The principal components (PCs) were extracted from the matrix through a method called singular value decomposition that realigns the data into the direction of the maximum variation^21^. PCs are uncorrelated and displayed according to the amount of variance explained - known as eigenvalue. The first PC (PC1) explains more of the total variance, followed by PC2, PC3, and so forth^22^. A lack of correlation means that the PCs are measuring different features. Interpretation of the components is based on the contribution of the variables to each PC^23^. In the present study, only the gait parameters whose contribution was equal to or greater than -/+ 0.40 were considered in the composition of each PC^21^.

The structure of the PCs combined with the individual standardized variables results in the subjects' scores, which represent the distance each individual is from the mean of one specific component^22^. The resultant PC scores were submitted to a linear discriminant analysis to determine which PCs could discriminate the groups.

In addition, to better understand the relation among the gait parameters and the behavior of the groups in relation to each other and to the gait parameters, a PCA-biplot was built. The PCA-biplot has its axes represented by the first two components, with maximum variance - PC1 and PC2. Interpretation of the biplot involves observing the length, proximity, and direction of the variables' vectors. The length of each vector approximates the amount of variance in each original variable that is captured by the 2-PC model; where longer vectors indicate higher variance^24^. The angle between two variables' vectors represents their pairwise correlation - the closer the vectors are to each other, the higher their correlation^25^. The direction of the variables' vectors with respect to the axes indicates the PC to which each variable is most strongly related.

Another important characteristic that can be extracted from the PCA-biplot is the spatial proximity or distance of the groups in relation to each other and to a set of variables, which reflects their similarities. When the perpendicular projection of the groups falls in the direction of the variables' vectors (solid line), the average value for the groups for these specific variables is higher. If the projection falls in the opposite direction of the vectors, i.e. on the extension of the vectors (dotted line), the average value for the groups for these specific variables would be lower. In addition, the projection on the variables' vectors that shows higher distance between the groups could be interpreted as the most important variable in group separation.

## Results

Of the 148 participants who entered the study, 133 (89.9%) completed the follow-up period. The dropout rate was 10.1%: 7 elderly women refused to continue participation in the study, 3 had health issues, 1 died, and 4 participants were excluded due to missed phone contacts over a 3-month period. There were no significant differences in the descriptive characteristics and gait parameters between those who lost the follow-up and those included in the analyses.

The one year prospective monitoring period showed a total of 23 (17.3%) recurrent fallers with a range of 2 to 9 falls and 110 (82.7%) non-recurrent fallers (40 one-time fallers and 70 did not fall). Overall, 108 falls were recorded, of which 68 (63%) resulted in injuries. The most severe injuries reported included one knee joint dislocation and three fractures (arm, nose, and wrist). Falls occurred more often outside their home (55.6%), while walking on the street or sidewalk (25.9%). Tripping was the most common perceived cause of the falls (45.4%).

Baseline participants' characteristics are summarized in Table 1. A significantly higher proportion of the participants in the recurrent fallers group (39%) reported two or more falls in the year prior to the beginning of the study compared to the non-recurrent fallers group (14%; p=0.007). No other significant differences were observed between the groups with regard to descriptive characteristics. Table 2 displays the spatiotemporal gait parameters for total sample and both groups.

**Table 1:** Descriptive characteristics of the participants in the beginning of the study.

**Characteristics**	**All participants (** *n* **=133)**	**Recurrent Fallers (** *n* **=23)**	**Non-recurrent Fallers (** *n* **=110)**	*p*-**value**
Sociodemographic and clinical				
Age (years), mean±SD	71.6±4.8	73.2±5.3	71.2±4.6	0.1^b^
Education (years), mean±SD	6.4±4.8	6.5±5.1	6.4±4.8	0.8^b^
BMI (kg/m^2^), mean±SD	27.2±4.4	26.4±4.3	27.4±4.4	0.3^a^
Total medication (number), mean±SD	3.0±2.1	4.0±2.9	2.8±1.8	0.1^b^
Chronic condition (number), mean±SD	2.6±1.8	3.3±2.5	2.4±1.6	0.2^b^
Hypertension, n (%)	93 (70)	18 (78)	75 (68)	0.5^c^
Diabetes, n (%)	17 (13)	2 (9)	15 (14)	0.7^c^
Osteoarthritis, n (%)	30 (23)	4 (17)	26 (24)	0.6^c^
Osteoporosis/Osteopenia, n (%)	36 (27)	7 (30)	29 (26)	0.8^c^
Physical activity^†^, n (%)	97 (73)	14 (61)	83 (75)	0.2^c^
Falls in previous year (≥ 2 falls), n (%)	24 (18)	9 (39)	15 (14)	0.007^c *^
Psychological, cognitive, and dynamic balance				
Fear of falling (Yes), n (%)	60 (45)	8 (35)	52 (47)	0.4^c^
FES-I (score), mean±SD	22.9±5.9	24.8±7.0	22.5±5.6	0.2^b^
MMSE (score), mean±SD	27.1±2.8	26.5±3.3	27.3±2.7	0.5^b^
TUG (s), mean±SD	8.3±0.9	8.4±0.9	8.3±0.8	0.6^a^

SD=standard deviation; BMI=body mass index; FES-I=Falls Efficacy Scale-International (range 16-64); MMSE=Mini-Mental State Examination (range 0-30); TUG=Timed up and go

a Independent *t* test significant at *p*<0.025

b Mann-Whitney test significant at *p*<0.008

c Chi-square test significant at *p*<0.05

* Statistical significance

† Aerobic or strength exercises two or more times a week.

**Table 2:** Mean±standard deviation of the spatiotemporal gait parameters of all participants and for recurrent fallers and non-recurrent fallers groups.

**Gait parameters**	**All participants (** *n* **=133)**	**Recurrent Fallers (** *n* **=23)**	**Non-recurrent Fallers (** *n* **=110)**
Velocity (cm/s)	127.9±15.6	125.8±15.9	128.3±15.6
Cadence (steps/min)	120.6±7.7	119.4±9.0	120.8±7.5
Step length (cm)	63.6±5.9	63.2±6.5	63.7±5.8
Base of support (cm)	7.5±2.6	8.0±2.8	7.4±2.5
Swing time (s)	0.388±0.03	0.391±0.03	0.388±0.03
Stance time (s)	0.611±0.04	0.620±0.05	0.609±0.04
Double support time (s)	0.225±0.03	0.231±0.03	0.224±0.03

PCA resulted in three components that explained 88.3% of the data variance. The loading vectors presented on Table 3 show the contribution of each gait parameter to the PCs, with the accumulated percentage of the total variance. PC1 had higher contribution from velocity, cadence, and stance time. PC2 had similar contribution from the variables step length and swing time and PC3 was heavily loaded with base of support.

**Table 3:** Loading vectors showing the contribution of the gait parameters to each principal component and the accumulated percentage of total variance.

**Gait parameters**	**Loading vectors**		
	**PC1**	**PC2**	**PC3**
Velocity (cm/s)	–0.46^†^	0.24	0.24
Cadence (steps/min)	–0.47^†^	–0.32	–0.09
Step length (cm)	–0.28	0.55^†^	0.38
Base of support (cm)	0.06	–0.35	0.86^†^
Swing time (s)	0.33	0.55^†^	0.04
Stance time (s)	0.49^†^	0.12	0.12
Double support time (s)	0.35	–0.28	0.13
Cumulative percentage of total variance (%)	53.7	74.5	88.3

PC=principal component

† Principal components were composed only by the gait parameters whose loading vector was equal to or greater than -/+ 0.40

Linear discriminant analysis with a stepwise procedure was conducted with the three components. The covariance matrices were similar between groups (p=0.304); however, the Wilks' lambda test was not statistically significant (p>0.05), indicating that none of the three components could discriminate the recurrent fallers group from the non-recurrent fallers group.

**Figure 1:**
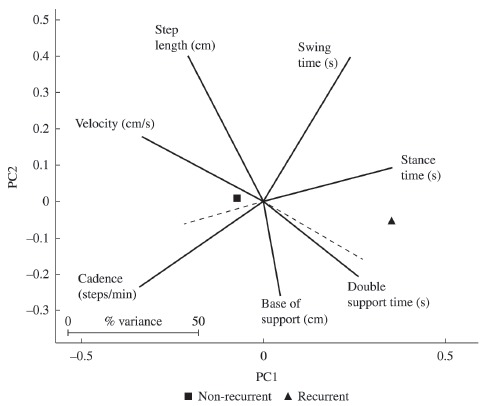
PCA-biplot with the average scores from the recurrent fallers and non-recurrent fallers groups.

Following, the PCA-biplot shown on Figure 1 was built with the x-axis represented by PC1 and the y-axis by PC2, the first two components that carry most of the explained variance. The average score of each group is represented by symbols and the gait parameters by vectors. The configuration of the PCA-biplot shows that most of the variables are well represented in the PC1 and PC2 dimensions with base of support showing the shortest vector. The highest relative variance is attributed to step length and swing time with the longest vectors. The variables velocity and step length showed the highest correlation due to the closest proximity.

The projection of the average score of the recurrent fallers group onto the variables' vectors shows that recurrent fallers walked with lower velocity, cadence, and step length and increased swing and stance time. The projection of the non-recurrent fallers group on the vectors shows the opposite pattern: higher velocity, cadence, and step length and lower swing and stance time. The highest distances between the groups are along the vectors of the variables velocity and stance time; therefore, these two variables or PC1 are important for group separation. PC1 had higher contribution from the variables velocity and cadence, with the vectors going in a negative direction, and from stance time with the vector going in a positive direction. Therefore, PC1 is a measure of rhythm since a decreased velocity and cadence and increased stance time would decrease rhythm and the opposite would increase the gait rhythm.

For a thorough interpretation of the behavior of the groups in relation to the dimension rhythm or PC1, Table 4 was built with the coefficients from PC1 represented only by signs, positive (+) or negative (-), according to the direction of the variables' vectors. In addition, in order to simplify interpretation, when the projection of the average group score fell in the direction of the variables' vector (solid line), one plus (+) sign was assigned, indicating high value and when the projection fell on the extension of the vector (dotted line), a negative (-) sign was assigned, indicating low value. The objective was to interpret the results of the biplot within the structure of PC1. The results showed that recurrent fallers tend to decrease their rhythm when walking and non-recurrent fallers do the opposite, increase their rhythm. Although the statistical test failed to show group discrimination, the interpretation from the PCA-biplot shows a trend from the recurrent fallers to decrease the gait rhythm by decreasing velocity and cadence and increasing the time spent in stance.

**Table 4:** PC1 loading vectors direction and the result of the projection from each group's average score onto the variables' vectors on the PCA-biplot.

**Gait parameters**	**PC1**	**RF**	**NRF**
Velocity (cm/s)	-	-	+
Cadence (steps/min)	-	-	+
Stance time (s)	+	+	-
Interpretation of the component	Rhythm		

PC1=first principal component; RF=recurrent fallers group; NRF=non-recurrent fallers group. For the PC1, the signs (+) and (-) represent the direction of the variables' vectors, while for the RF and NRF groups, the sign (+) indicates high value and (-) low value.

## Discussion

The aging world population is characterized by a predominance of females^26^. Additionally, when compared to men, women have a higher risk of falling^1^. Factors such as genetics, lifestyle, longevity, and frailty have been pointed out as potential reasons for the increased risk of falls among women^27^. Therefore, it is essential to deliver appropriate support to this vulnerable group. The aim of this study was to investigate, by means of PCA and discriminant analysis, whether gait velocity and other spatiotemporal gait parameters could predict recurrent falls in a sample of community-dwelling elderly women. Since gait parameters are highly correlated^23^, the use of multivariate statistic techniques is appropriate for examining the independent effect of these parameters on the likelihood of recurrent falls. Furthermore, the PCA-biplot was introduced to clarify interpretation of the principal components^28^.

Our results showed that neither gait velocity nor the other gait parameters significantly predicted recurrent falls. These results are consistent with another prospective study that also found that none of the gait variables could predict recurrent falls^12^. In a 1-year follow-up conducted in 97 healthy active women (68.73±7.07 years), Paterson et al.^12^ observed that none of the spatiotemporal gait parameters (velocity, stride length, foot angle, base of support, stride time, stance time, and swing time) differed significantly between multiple fallers (two or more falls) and non-multiple fallers.

Conversely, one recent prospective population-based study that used the same gait analysis system as the present study found an association between spatiotemporal gait parameters and the risk of recurrent falls^29^. Callisaya et al.^29^ observed in 412 elderly individuals (both sexes, 60-86 years) that gait velocity and cadence were associated in a nonlinear way with the risk of multiple falls. The spatiotemporal gait parameters were divided into quarters, and the results for gait velocity showed that the participants in the second quarter (velocity between 1.02 and 1.16 m/s) showed a lower risk of suffering recurrent falls. These results indicate that a gait velocity greater than 1.02 m/s protects against recurrent falls, but this protective effect is reduced when gait velocity exceeds 1.16 m/s. According to the authors, this reduction in protection occurs because some elderly individuals walk too fast for their physical capabilities or participate in high-risk physical activities, which places them at a higher risk of falling. Furthermore, they observed that greater variability in step length and double support phase was independently and linearly associated with increased risk of multiple falls. Thus, this result suggests that gait variability measures may be more sensitive in predicting recurrent falls than more conventional gait parameters, such as velocity.

The population-based study described above adopted broader selection criteria, including participants with health conditions that affect mobility and gait, such as dementia, stroke, and Parkinson's disease^29^. Thus, the inclusion of participants with walking disabilities and the differences between participants in characteristics like sex may explain the different results between the present study and the study by Callisaya et al.^29^.

Our study has several strengths. The prospective record of falls and the biweekly contact with participants minimized the potential occurrence of recall bias. This study had low attrition rate for the follow-up (10.1%), and the participants who completed and did not complete the follow-up were similar. In addition, we adopted rigorous eligibility criteria, resulting in a homogenous sample with well-defined characteristics. The participants in our sample were relatively young (average age=71.6), did not exhibit cognitive impairments, had few comorbidities, used very little medication, and had excellent dynamic balance, as demonstrated by their performance on the TUG test^30^. Furthermore, compared to gait normative data of healthy and robust elderly women of the same age group^31^, our participants performed better in most of the spatiotemporal gait parameters. Considering the sample profile and the circumstances by which the majority of falls occurred, it can be inferred that other factors minimally related to gait contributed to the number of falls amongst our participants. These factors include environmental risks and degree of exposure to situations that present an inherently high fall risk. Therefore, our results are meaningful because they reveal that healthy elderly women without mobility dysfunction may also fall, but spatial and temporal gait data are not predictive of those falls.

In this study, the groups of elderly women were similar in all evaluated descriptive characteristics, except for their history of falls. A significantly higher proportion of the participants in the recurrent fallers group reported two or more falls during the year prior to the study compared to non-recurrent fallers. Although this information is retrospective and subject to recall bias, it was previously demonstrated that a history of two or more falls in the previous year is an important risk factor for recurrent falls in the community-dwelling elderly^32^. Therefore, our results support existing knowledge and indicate that a history of recurrent falls should be considered in the assessment of elderly individuals, since those who fall in a recurrent manner have a higher chance of falling multiple times in the following year.

In recent years, statistical approaches, such as PCA, have been used to analyze gait data^23^^,^ 33. One of the major challenges of this analysis is its interpretation. In this study, the biplot technique was used to explore the contribution of the gait variables to the components^23^. The biplot revealed that the groups are very far apart in relation to gait velocity and stance time. Previous studies demonstrated the importance of these gait parameters in differentiating elderly recurrent fallers from non-recurrent fallers^6^^,^ 11. Further interpretation of PC1 revealed that the variables' contribution described gait rhythm. Recurrent fallers tend to slow their rhythm when they walk, while non-recurrent fallers exhibit an opposite walking pattern characterized by an increased gait rhythm. Although group discrimination is not statistically significant in the observations, these trends cannot be rejected. Clinically, these trends could be considered as red flags - an indication that elderly individuals in these conditions may be more susceptible to recurrent falls. Therefore, the biplot was able to capture a tendency towards group separation and identify an underlying pattern (rhythm) that could not be seen when applying traditional statistical tests.

There are certain limitations to this study. Since sample selection was based on convenience, the generalizability of the findings is limited. Second, gait variability was not measured in our study; thus, not all possible aspects of gait that potentially contribute to recurrent falls in the elderly were studied. Finally, gait was tested at the usual pace, which might not have been sensitive enough to reflect the recurrent fall risk of participants. Future studies should investigate the capacity for gait parameters to predict recurrent falls in healthy elderly individuals without mobility impairment in "real life" situations, such as walking over obstacles or performing cognitive and motor tasks (e.g. speaking, making calculations or carrying objects). It is possible that more challenging tasks may place greater demand on physiological and cognitive systems and therefore be more informative about recurrent falls risk.

## Conclusions

The analyzed spatiotemporal gait parameters failed to predict recurrent falls in this group of healthy, community-dwelling 65-85 year-old elderly women without mobility impairment. However, the PCA-biplot technique revealed tendencies in group separation and differences in walking patterns between groups. These findings could be used as red flags suggesting that further investigation into the gait of elderly patients should be considered.
